# Role of Ni Layer Thickness in Regulating Mechanical Properties and Deformation-Fracture Behavior of TiB_2_-Ni Multilayer Films

**DOI:** 10.3390/nano15221687

**Published:** 2025-11-07

**Authors:** Xiaoben Qi, Xu Wang, Lina Tang, Rukeye Maimaititaji, Miaoling Shi, Sinan Ding, Jianyuan Ma, Huanqing Xu, Jinyi Fan, Hailong Shang, Ying Wang

**Affiliations:** 1 School of Materials Science, Shanghai Dianji University, Shanghai 201306, China; qixb@sdju.edu.cn (X.Q.); wx19980927@163.com (X.W.); 19301363956@163.com (R.M.); sml10s0@163.com (M.S.); 19855328893@163.com (S.D.); 16691686060@163.com (J.M.); hh369792@126.com (H.X.); fjy060510@outlook.com (J.F.); 2 Shanghai Aerospace Equipments Manufacturer Co., Ltd., Shanghai 200240, China; linatang149@163.com

**Keywords:** TiB_2_-Ni multilayer, microstructure, mechanical properties, strengthening and toughening, deformation-fracture mechanisms

## Abstract

A series of TiB_2_-Ni multilayer films with different Ni layer thicknesses was prepared by magnetron sputtering technology. The effect of Ni layer thickness on the microstructure and mechanical properties of the multilayer films was investigated, and the deformation and fracture mechanisms underlying the observed behavior were analyzed in detail. The results show that all multilayer films exhibit a well-defined layered architecture with sharp interfacial boundaries. Specifically, the Ni layers grow as columnar grains with an average diameter of approximately 10 nm, while the TiB_2_ layers form a very fine acicular nanocolumnar structure. With the increase in Ni layer thickness, the hardness of the multilayer films shows a decreasing trend, gradually decreasing from 27.3 GPa at a 4 nm Ni thickness to 19.3 GPa at 50 nm. In contrast, the fracture toughness increases gradually from 1.54 MPa·m^1/2^ to 2.73 MPa·m^1/2^. This enhancement in toughness is primarily attributed to a transition in the deformation and fracture mechanism. With the increase in Ni layer thickness, the crack propagation mode in the multilayer films gradually changes from the integral propagation penetrating the film layers to the crack deflection propagation within the layers. This transformation is the result of the combined effect of the stress state of each layer and the crack energy dissipation.

## 1. Introduction

Due to its unique structural characteristics, TiB_2_ exhibits numerous outstanding physicochemical properties, including high hardness, high melting point, and excellent electrical and thermal conductivity. These exceptional properties arise from its distinctive crystal structure and the synergistic interaction between Ti-B ionic bonds and B-B covalent bonds [1]. However, the inherent brittleness and poor toughness of TiB_2_ ceramic films result in high residual stresses after deposition, limiting their widespread application. Consequently, the development of TiB_2_ thin-film materials with superior comprehensive properties has become a current research focus [2,3,4].

Regarding the enhancement of coating toughness, two primary technical approaches currently exist. The first approach involves the addition of toughening phases (such as metallic elements Ni, Al, Cu, or non-metallic elements N, C, Si) to improve the overall performance of the coating by forming a special ceramic/toughening phase composite structure [5,6,7]. For example, Ji et al. [8] investigated the effect of Cu doping on the microstructure and properties of TiB_2_ coatings, demonstrating that Cu enhances toughness by absorbing shear stress energy through plastic deformation and inhibiting crack propagation. Wang et al. [9] observed that Ni-doped TiB_2_ forms a unique ceramic-metal nanocomposite structure, similarly improving coating toughness. However, this approach heavily depends on the content of added elements for composite structure formation, presenting challenges in precise control.

The second approach mimics natural biomaterials such as seashells by designing multilayer structures to enhance film toughness. Such nanostructured multilayer films, often referred to as nanolaminates, are characterized by their alternating layered structure with individual layer thicknesses in the nanoscale regime. This architecture combines the advantages of both material classes, simultaneously achieving high hardness and high toughness with simple, easily controllable structures [10,11,12,13,14]. Extensive research has also been conducted on TiB_2_-based nanolayer films. For instance, Wolfe et al. [13] observed that residual stresses in multilayer TiB_2_/TiC coatings decrease with increasing layer count, thereby improving coating toughness. He et al. [14] prepared TiB_2_/a-C nanolayer films with modulated periods ranging from 1 to 10.5 nm via magnetron sputtering, confirming significant enhancements in both hardness and toughness.

However, most of the above studies have focused on investigating the influence of layered structure parameters on the mechanical properties of nanolaminar films. The deformation mechanism of TiB_2_-based nanolaminar films, particularly the understanding of how layered structure parameters regulate the fracture mechanism of multilayer films, remains insufficiently explored. This study employs the TiB_2_-Ni system as its research subject, preparing a series of TiB_2_-Ni nanolayer films with varying Ni layer thicknesses. It investigates the influence of Ni layer thickness on the microstructure and mechanical properties of the multilayer films and discusses the deformation-fracture mechanisms of the multilayer films under different Ni layer thicknesses.

## 2. Materials and Methods

### 2.1. Film Deposition

In this experiment, TiB_2_ films with varying Ni layer thicknesses were deposited on single-crystal silicon substrates (10 mm × 10 mm × 1 mm) using a magnetron sputtering system (ANAVA SPC-350, Anelva, Tokyo, Japan). The silicon substrates were ultrasonically cleaned with acetone for 10 min. After drying, the substrate was mounted on a holder within the sputtering chamber. The system was operated using dual RF power supplies: one set at 150 W for pure TiB_2_ targets (99% purity, Φ76 mm) and the other at 20 W for pure Ni targets (99% purity, Φ76 mm). The chamber was evacuated to a base pressure of 2 × 10^−3^ Pa before introducing high-purity argon (99.99%) as the sputtering gas. During deposition, the working pressure was maintained at 0.5 Pa.

The fabricated materials consisted of TiB_2_-Ni multilayered thin films, with alternating TiB_2_ and Ni layers deposited sequentially via direct sputtering. A Ti interlayer was initially deposited (using a DC power supply at 0.5 A, 20 rpm for 10 min) to enhance film adhesion. This Ti layer is crucial for improving the bonding between the native silicon oxide layer on the substrate and the subsequent TiB_2_-Ni multilayer, as the reactive Ti metal can form a chemically strong interface with the SiO_2_ surface and act as a stress-relaxing layer. The TiB_2_ layer deposition time was fixed at 10 min, while the Ni layer deposition times were varied at 0.5, 1, 2.5, 5, and 10 min to produce five distinct multilayer structures. Each film group underwent a total deposition time of 1 h. The depositions were carried out without applying substrate bias and at room temperature.

### 2.2. Film Characterization

The cross-sectional morphology of the sputtered films was examined using a scanning electron microscope (ZEISS Sigma 300, ZEISS, Oberkochen, Germany), and cross-sectioned thin sections prepared by focused ion beam (FIB) were analyzed using a transmission electron microscope (JEM-2010ARP, JEOL, Tokyo, Japan). Selected-area electron diffraction patterns, obtained using a ϕ150 nm aperture, were employed for phase analysis. The mechanical properties were evaluated via nanoindentation using a 300-NTh3 instrument (Anton Paar, Graz, Austria) for TiB_2_ films with varying Ni layer thicknesses. A force of 10 mN was applied to ensure the indentation depth did not exceed the total film thickness. The loading and unloading durations were set to 30 s, with a holding time of 10 s. To minimize measurement error, eight replicates were performed for each sample. The load–displacement curves were analyzed using the Oliver-Pharr method [15] to determine the hardness (H), elastic modulus (E), maximum indentation depth (hmax), and residual indentation depth (hp) of the films. The toughness of the films was assessed by calculating parameters such as H/E, H^3^/E^2^, and the normalized plastic depth value (δ_H_). For further evaluation of film toughness, the Vickers indentation method was employed, applying a load of 2 N to the film surface. Following indentation, The fracture toughness was calculated using Equation(1) [16] based on the crack length.

(1) $$K_{IC}=\delta \;{{\left(\frac{a}{l}\right)}^{\frac{1}{2}}(\frac{E}{H})}^{\frac{2}{3}}\frac{P}{c^{3/2}}$$

where K*_IC_*: Fracture toughness (MPa·m^1/2^), δ: Empirical coefficient of 0.016 [17], E: Elastic modulus of films (GPa), H: Hardness of the films (GPa), P: Indentation load (mN), a: The length from the center to the corner of the indentation (µm), l: The length of the crack (µm), c: the total crack length (µm), which is given by the sum of a and l.

The nanoscratch tests were performed on Tribology Test Equipment (CETR UNMT-1, CETR, Campbell, Camden, NJ, USA). A diamond tip with a radius of 2.5 µm was used to generate a series of 2 mm-long wear tracks on the film surface at a constant velocity of 12.5 µm/s, with the load increasing linearly from 2 mN to 150 mN. Surface roughness measurements of scratch morphology were performed using an optical profilometer (Leica DCM 3D, Wetzlar, Germany).

## 3. Results

### 3.1. Microstructure

**Figure 1:**
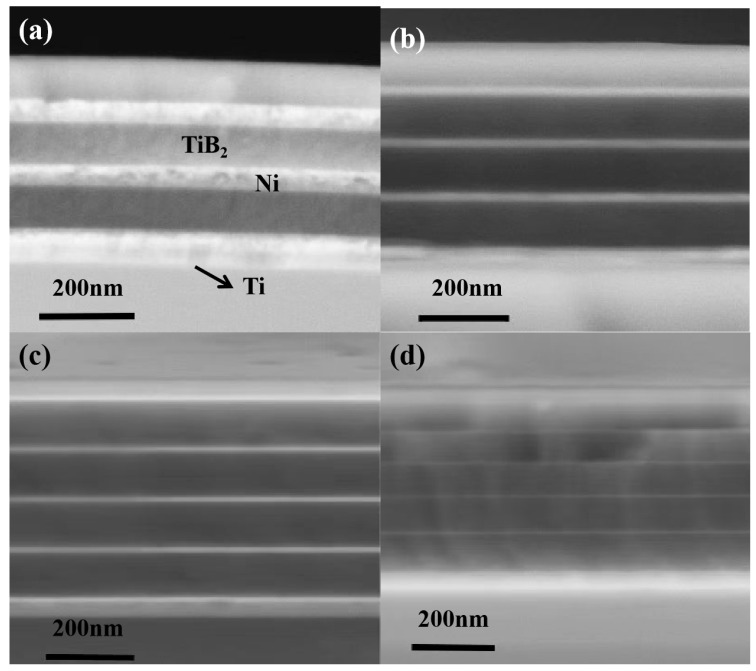
SEM images of multilayers with different thicknesses of Ni layer (**a**) 50 nm; (**b**) 25 nm; (**c**) 16 nm; (**d**) 4 nm.

To elucidate the role of the metallic layer, a series of TiB_2_-Ni multilayer films was fabricated with systematically varied nickel (Ni) layer thicknesses. As summarized in Table 1, the TiB_2_ layer thickness was held constant at 100 nm across all samples, while the Ni layer thickness was precisely tuned from 4 nm to 50 nm. Figure 1 presents the cross-sectional morphology of the TiB_2_-Ni multilayer film. As shown in the figure, the film exhibits a well-defined laminated alternating structure, characterized by distinct interfaces and consistent thickness between layers. The titanium layer is first deposited on the substrate surface to enhance the bonding strength between the films and the substrate. The bright white layers correspond to the nickel layer, whereas the dark layers represent the titanium diboride (TiB_2_) layer.

**Figure 2:**
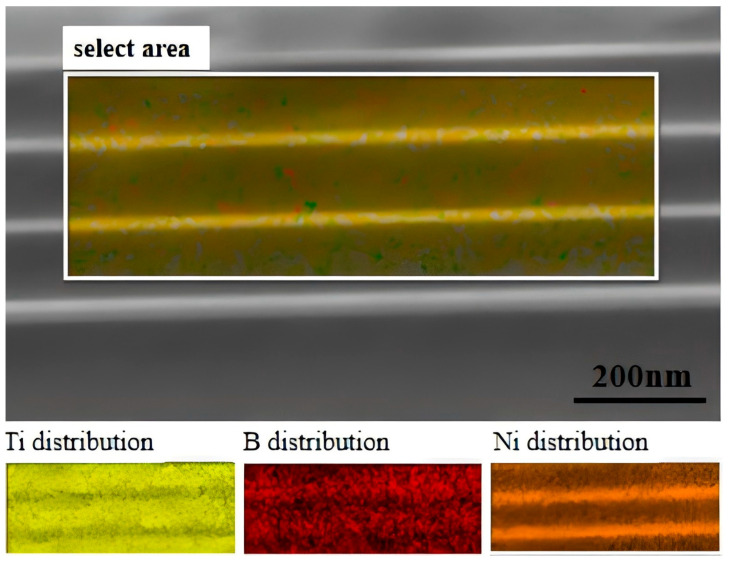
Cross-sectional EDS mapping analysis of the multilayer film with a Ni layer thickness of 25 nm.

To further verify the elemental distribution and interfacial characteristics of the multilayer film, cross-sectional EDS mapping analysis was performed on a sample with a Ni layer thickness of 25 nm, as shown in Figure 2. The selected area image clearly reveals the layered architecture of the film. The elemental distribution maps for Ti, B, and Ni displayed below are in precise spatial correspondence with the layered structure: Ti (yellow) and B (red) signals colocalize within the thicker layers, confirming the presence of the TiB_2_ phase, whereas the Ni signal (brown) is strictly confined to the thinner, alternating layers. Notably, elemental profiles across the interfaces exhibit sharp concentration gradients and well-defined boundaries, with no significant interdiffusion observed. This result provides direct evidence of strong interfacial adhesion and high elemental selectivity between the TiB_2_ and Ni layers, resulting in the formation of a structurally ordered multilayer heterostructure, which is essential for enabling the interface toughening mechanism.

**Figure 3:**
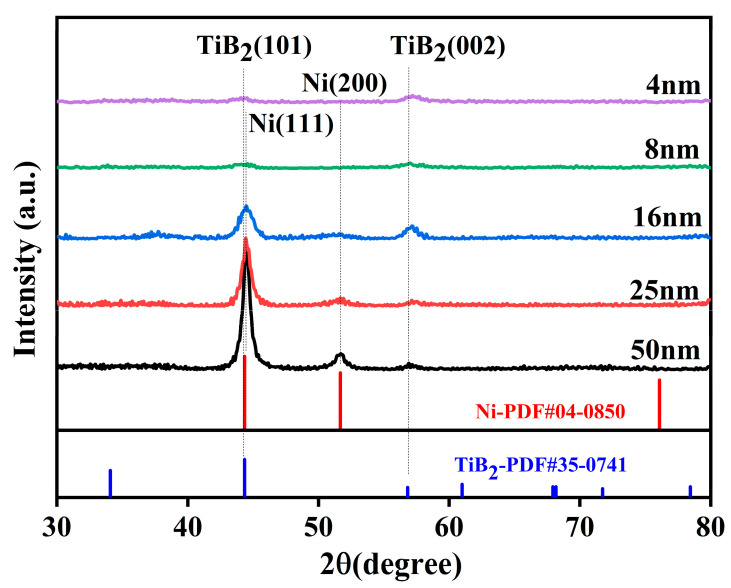
XRD patterns of TiB_2_-Ni multilayer films with different Ni layer thicknesses.

XRD analysis of TiB_2_-Ni multilayer films with varying Ni layer thicknesses is presented in Figure 3. All samples exhibit similar diffraction peaks, featuring three prominent peaks at approximately 44.45°, 51.85°, and 57°. The most intense peak near 44.45° arises from the overlapping contributions of the TiB_2_ (101) and Ni (111) reflections. This assignment is consistent with the reference patterns for TiB_2_ (PDF#35-0741) and Ni (PDF#04-0850), confirming that both phases possess a polycrystalline nature. The additional peaks at 51.8° and 57° are attributed to the Ni (200) and TiB_2_ (002) crystal planes, respectively. With increasing nickel layer thickness, the contribution from the nickel (111) crystal plane becomes increasingly dominant around 44.45°. Concurrently, the diffraction peak corresponding to Ni (200) grows more pronounced as the Ni layer thickness increases. These observations indicate that during the growth of the multilayer film, the Ni layer develops a strong (111) fiber texture, and this preferred orientation becomes increasingly pronounced with greater layer thickness. As the thickness of the Ni layer increased from 4 nm to 50 nm, the full width at half maximum of the Ni (111) diffraction peak significantly decreased. This indicated that the grain size of the Ni layer significantly increases with the increase in layer thickness. The intensity and width of the TiB_2_ (002) diffraction peak did not undergo significant changes, suggesting that the crystal structure of the TiB_2_ layer is less affected by the change in the thickness of the Ni layer.

**Figure 4:**
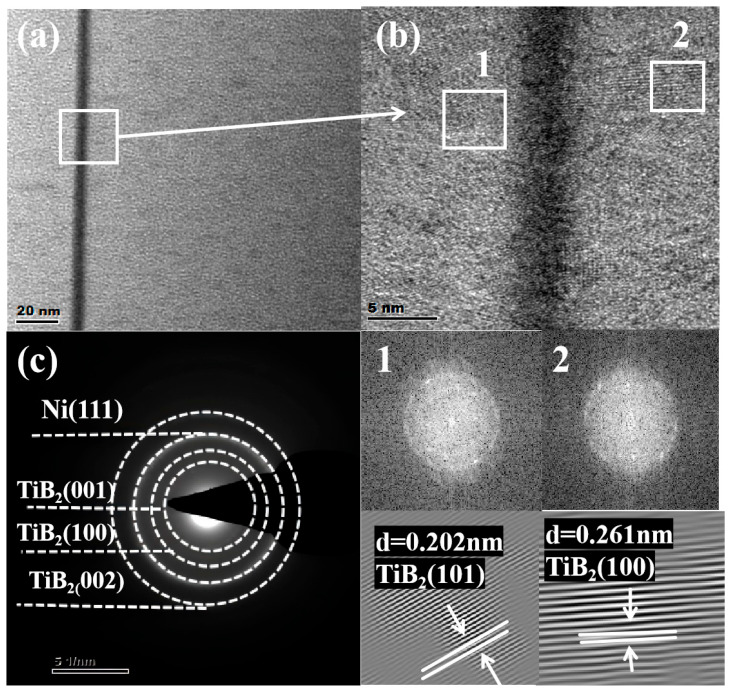
TEM image of multilayer film at 4 nm Ni layer thickness (**a**) Cross-section of low magnification bright field image; (**b**) Cross-section of a high magnification bright field image; (**c**) SAED drawing from the area marked as square in the figure.

The TEM analysis provided nanoscale evidence for the above XRD results. Figure 4 presents the low-magnification bright-field TEM image, selected electron diffraction pattern, and HRTEM image of the cross-sectional films when the thickness of the Ni layer is 4 nm. In the low-magnification bright-field image of Figure 4a, the TiB_2_-Ni multilayer coating retains a favorable layered structure, where the dark stripes represent the Ni layer and the light stripes represent the TiB_2_ layer. The interface is distinct and straight with low roughness. The coating grows in a columnar crystal mode along the direction perpendicular to the coating surface, and the columnar crystals penetrate the entire coating thickness. It can also be seen in Figure 4b that there are many lattice fringes existing as nanoclusters in the TiB_2_ layers. Figure 4c shows the corresponding electron diffraction pattern of the red-framed region in Figure 4a. It is quite evident that the TiB_2_-Ni multilayer coating is a polycrystalline structure. The SAED pattern presents a distinct crystalline diffraction ring corresponding to Ni (111) or TiB_2_ (101), TiB_2_ (100), and TiB_2_ (002). Both the Ni (111) and TiB_2_ (101) have almost the same interplanar distance of about 0.2 nm, which suggests a possible relationship between the lattice templates at the interface and the epitaxial growth process.

**Figure 5:**
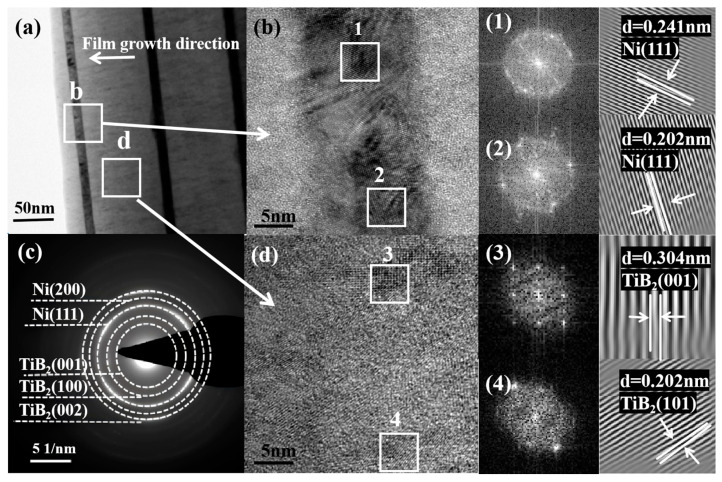
TEM image of multilayer film at 16 nm Ni layer thickness; (**a**) Cross-section of low magnification bright field image; (**b**) Cross-section of a high magnification bright field image; (**c**) SAED drawing from the area labeled square b; (**d**) High-resolution image of the bright field in the white square d region.

When the thickness of the Ni layer increased to 16 nm, the evolution of its microstructure became more pronounced, as given in Figure 5. From Figure 5a, it can be seen that the film forms a clear layer structure, where the thicker one is the TiB_2_ layer and the thinner one is the Ni layer, and the interlayer interface between the TiB_2_ layer and the Ni layer is very clear. The high-resolution image of Figure 5b and its Fourier transform spectrum clearly identify the Ni (111) crystal plane, indicating that the Ni layer in this area has good crystallinity. The corresponding SAED pattern of Figure 5c shows that the diffraction pattern at the interface of Ni and TiB_2_ layers. It is obvious that the TiB_2_-Ni multilayer coating is a polycrystalline structure. The SAED patterns show distinct crystal diffraction rings corresponding to Ni (111), Ni (200), TiB_2_ (001), TiB_2_ (100), and TiB_2_ (002). This result is consistent with the above XRD analysis. Figure 5d shows a local enlargement of the TiB_2_ layer, and the electron diffraction patterns obtained by Fourier transform of the regions 1 and 2 in Figure 5d show that this region exhibits a unique crystal structure, and the lattice distances of the TiB_2_ (001) and (101) planes are about 0.304 nm and 0.202 nm, respectively. The clear detection of the Ni (200) ring here, despite its weak signature in the bulk XRD data (Figure 3), confirms the presence of grains with this orientation. Its lower intensity in XRD is consistent with the strong (111) preferred growth texture of the Ni layers, as discussed earlier.

In the TiB_2_-Ni multilayer film, the TiB_2_ layer exhibits a stable nanoscale columnar crystalline structure, whereas the microstructure of the Ni layer demonstrates a strong dependence on its thickness. With increasing Ni layer thickness, the microstructure evolves progressively from initially fine nanocrystallites to coarser columnar grains, accompanied by marked improvements in crystallinity and (111) crystallographic texture. This microstructural evolution constitutes the fundamental structural basis for the significant changes observed in the mechanical properties, particularly in hardness and toughness.

### 3.2. Mechanical Properties

**Figure 6:**
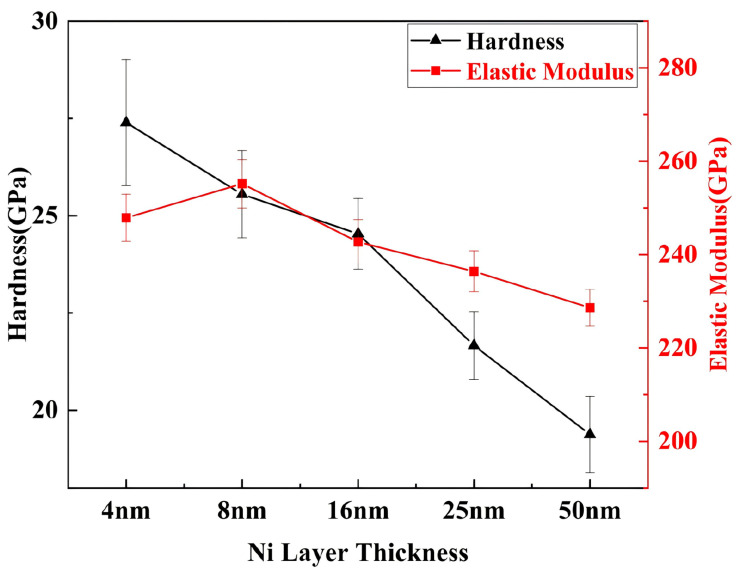
Hardness and elastic modulus of multilayer films with different nickel layer thicknesses.

Figure 6 presents the hardness and elastic modulus profiles of the multilayer films. As shown in Figure 6, the film exhibited a hardness of 27.3 ± 1.61 GPa when the Ni layer thickness was 4 nm, with a gradual decrease in hardness observed as the Ni layer thickness increased. The multilayer film reached a minimum hardness of 19.3 ± 0.97 GPa when the Ni layer thickness was 50 nm.

When the Ni layer thickness was relatively thin, the increase in the number of plating cycles resulted in a higher number of interfaces within the multilayer film. This led to increased lattice distortion at the interfaces and enhanced the pinning effect of dislocations, thereby increasing resistance to motion and consequently improving film hardness [18], thereby increasing resistance to motion and consequently improving film hardness. Conversely, as the Ni layer thickness increased, the proportion of soft-phase metals rose, reducing the number of coating cycles. Consequently, the interfacial strengthening effect diminished, leading to a decrease in coating hardness.

The elastic modulus increased from 247.9 ± 5 GPa to 255.2 ± 5.1 GPa when the Ni layer thickness was increased from 4 nm to 8 nm. However, as the Ni layer thickness was further increased to 50 nm, the elastic modulus gradually decreased to 228.6 ± 3.88 GPa.

**Figure 7:**
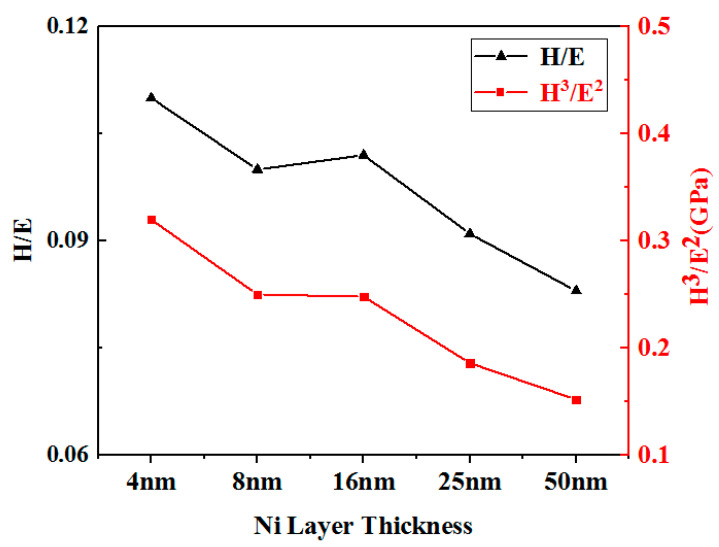
H/E, H^3^/E^2^ of multilayer films with different nickel layer thicknesses.

Figure 7 presents the pre-yield elastic strain (H/E) and resistance to plastic deformation (H^3^/E^2^) of TiB_2_-Ni multilayer films. The H/E and H^3^/E^2^ values serve as predictors of wear behavior and are regarded as critical indicators for evaluating film toughness. Hard coatings exhibiting H/E ratios exceeding 0.1 demonstrate superior elasticity, a key characteristic for developing tough hard coatings [19]. The data reveal that as the Ni layer thickness increases from 4 nm to 16 nm, the multilayer films maintain H/E ratios ≥ 0.1, indicating high elasticity. However, when the Ni layer thickness further increases from 16 nm to 50 nm, the H/E ratio exhibits a gradual decline to values < 0.1. This trend suggests that thicker Ni layers (where metallic properties dominate) render the multilayer films more prone to plastic deformation, while simultaneously diluting the high elastic modulus and brittle characteristics of TiB_2_. Consequently, both H/E and H^3^/E^2^ ratios decrease with increasing Ni layer thickness.

**Figure 8:**
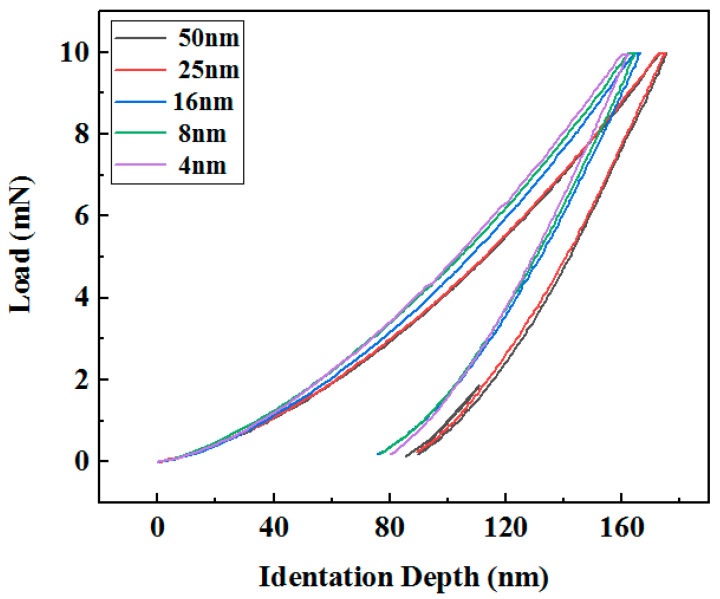
Load-indentation depth of multilayer films with different thicknesses of nickel layers.

Figure 8 presents the load-indentation depth curves for the multilayer films with different Ni layer thicknesses. These curves are fundamental for understanding the evolution of deformation behavior. A clear systematic shift in the curve morphology is observed. With increasing Ni layer thickness, the hysteresis loop enlarges and the residual indentation depth increases. This provides direct visual evidence of a transition from a more elastic recovery behavior (in films with thin Ni layers) to a more pronounced plastic deformation and energy dissipation character (in films with thick Ni layers).

In this experiment, the indentation depths ranged from 150 to 170 nm, exceeding one-tenth of the film thickness. When the indentation depth surpasses this threshold, the measured mechanical properties are inevitably influenced by the substrate [20]. As Korsunsky and A.M. [21] previously demonstrated, in cases where substrate effects cannot be avoided, a modified approach has been established to interpret the hardness of film systems—specifically, the concept of composite hardness arising from the interaction between the film and the substrate. Therefore, the reported values of hardness and elastic modulus should be understood as representing the combined response of the film–substrate system, rather than the intrinsic, substrate-independent mechanical properties of the film alone.

**Figure 9:**
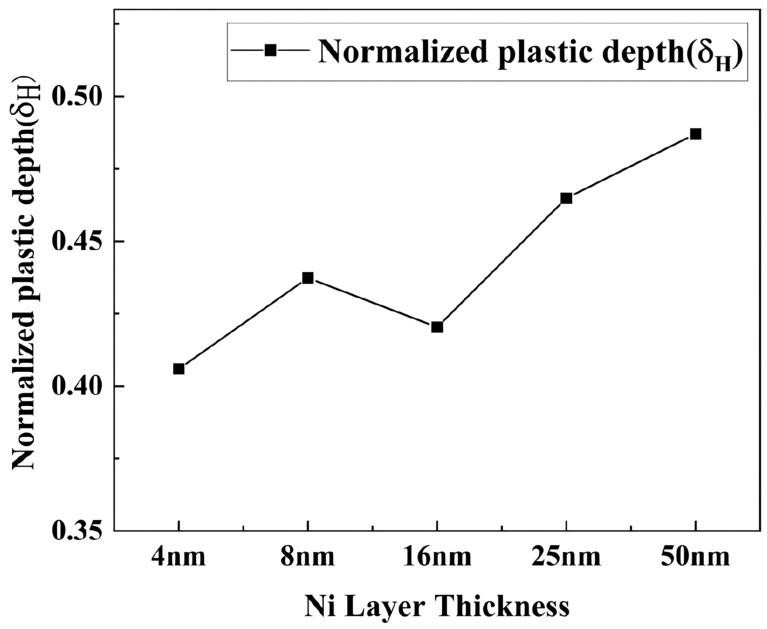
Normalized plastic depth values (δ_H_) for multilayers with different Ni layer thicknesses.

To quantify this observation, the normalized plastic depth *δ_H_* was derived from these curves, calculated as *δ_H_* = *h_p_*/*h_max_*, where *h_p_* represents the indentation depth at which no elastic deformation occurs and *h_max_* denotes the maximum indentation depth. The calculated *δ_H_* values are displayed in Figure 9.

The normalized plastic depth initially decreases and subsequently increases as the Ni layer thickness increases from 4 nm to 50 nm. When the Ni layer thickness increases from 4 nm to 16 nm, the number of cycles in the multilayer film decreases, leading to fewer interfaces and weaker interfacial regulation, thereby reducing the film’s plastic deformation capability. Conversely, when the Ni layer thickness increases from 16 nm to 50 nm, the plastic deformation capacity of the multilayer film significantly improves due to the higher Ni content.

**Figure 10:**
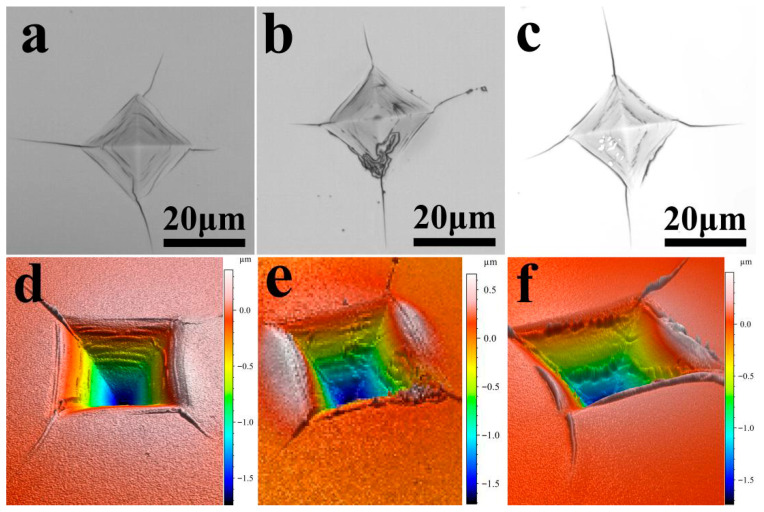
Indentation morphology of TiB_2_-Ni multilayer films with different Ni Layer thicknesses; (**a**) plan-view SEM micrographs and (**d**) 3D surface profiles of the indentations with a 4 nm Ni layer; (**b**) plan-view SEM micrographs and (**e**) 3D surface profiles of the indentations with a 16 nm Ni layer; (**c**) plan-view SEM micrographs and (**f**) 3D surface profiles of the indentations with a 50 nm Ni layer.

Figure 10 shows optical microscope images of indentations on TiB_2_-Ni multilayer films with different Ni layer thicknesses under a large load of 2 N. Among these images, Figure 10a–c present the plan-view morphologies of the indentations, while Figure 10d–f show the 3D morphologies. As observed from the figures, under a load of 2 N, cracks with different lengths appear at the four corners of the indentations. With the increase in Ni layer thickness, the length of the cracks shows a gradual decreasing trend, indicating an improvement in the toughness of the multilayer films. In addition, when the Ni layer thickness is 4 nm, there are many annular cracks surrounding the wall of the indentation pit (Figure 10a). Combining with the 3D morphology of the indentation in Figure 10c, it can be found that these annular cracks exhibit a stepped distribution. With the increase in Ni layer thickness, these stepped annular cracks gradually decrease. This phenomenon is related to the transformation of the deformation and fracture mechanisms of the multilayer films, and the specific reasons will be analyzed in the discussion section.

The formation and propagation of circumferential cracks primarily result from the macroscopic plastic deformation of both the substrate and the thin-film material. Consequently, the toughness of the thin film can be assessed by examining the circumferential cracks generated during indentation. When the nickel layer thickness is 4 nm, a greater number of annular cracks appear within the indentation, suggesting that the film absorbs more energy to accommodate deformation under loading. As the nickel layer thickness increases further, the number of annular cracks decreases, which is primarily associated with the higher metallic nickel content. The calculated fracture toughness values for the multilayer films are presented in Table 2. The data indicate that as the nickel layer thickness increases from 4 nm to 50 nm, the film toughness initially rises from 1.54 MPa·m^1/2^ to 1.70 MPa·m^1/2^, then decreases to 1.54 MPa·m^1/2^, and finally increases to 2.73 MPa·m^1/2^. As the thickness of the Ni layer increases, the XRD patterns show progressively narrower and more intense Ni diffraction peaks, indicating substantial grain growth. The enlarged grains offer increased space for dislocation glide, facilitating plastic deformation and thereby enhancing the overall toughness of the material.

**Figure 11:**
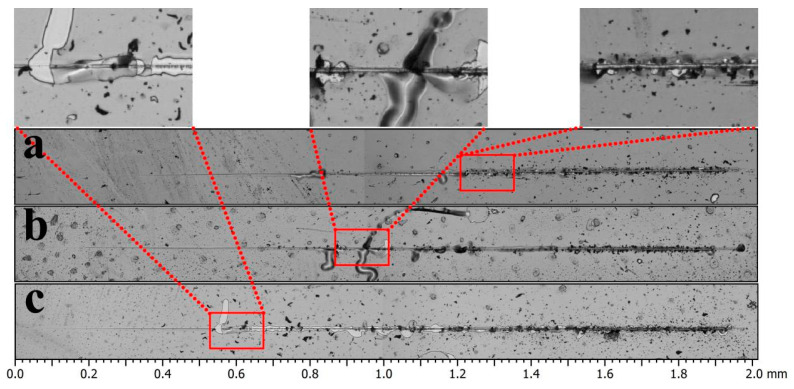
The nanoscratch morphology of multilayer films; (**a**) Ni layer thickness of 50 nm; (**b**) Ni layer thickness of 16 nm; (**c**) Ni layer thickness of 4 nm.

The toughness of the TiB_2_-Ni multilayer film was further evaluated through nano-scratch testing. Figure 11 presents the scratch morphology of multilayer films with varying nickel layer thicknesses, arranged from top to bottom: 50 nm, 16 nm, and 4 nm. As illustrated, the multilayer film with a 4 nm Ni layer exhibited delamination at approximately 0.5 mm (Figure 11c), accompanied by a relatively extensive delaminated area. When the Ni layer thickness increased to 16 nm, delamination occurred only at around 0.9 mm (Figure 11b), with a substantially reduced delaminated area. Further increasing the Ni layer thickness to 50 nm delayed delamination beyond 1.2 mm (Figure 11a). Local magnification reveals that most delaminations were confined to minimal areas. These findings indicate that increasing the Ni layer thickness delays crack initiation, reduces both crack propagation and delamination area, and consequently enhances the multilayer film’s toughness.

## 4. Discussion

### 4.1. Interface

**Figure 12:**
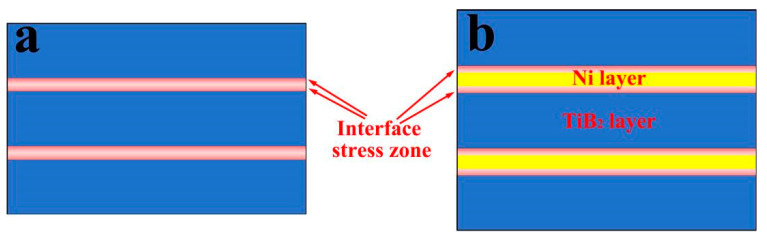
The schematic diagram of the distribution of interface stress zones in TiB_2_-Ni multilayer films with different Ni layer thicknesses; (**a**) thin nickel films; (**b**) thick nickel fims.

As a unique characteristic of layered materials, interfacial bond strength significantly influences their performance. High-strength interfacial bonding not only enhances the material’s overall strength but also improves toughness by regulating deformation mechanisms and crack propagation patterns. Excellent wettability serves as the foundation for achieving high-strength interfacial bonding. Research by Xi et al. [22] demonstrated that in a TiB_2_-Ni wetting system at 1500 °C, the contact angle of Ni on the TiB_2_ surface is only 32°. Consequently, the TiB_2_-Ni interface exhibits high bonding strength. This robust interface not only efficiently transmits loads but also generates an interfacial stress zone during deposition due to differing thermal expansion coefficients. This stress zone induces tensile stress in the Ni layer and compressive stress within the TiB_2_ layer. Figure 12 illustrates the distribution of the interfacial stress zone in multilayer films with varying Ni layer thicknesses. When the Ni layer is thick, the stress influence zone covers only part of the Ni layer. As the Ni layer thickness decreases, this stress zone may cover the entire Ni layer. The presence of this stress zone not only impedes dislocation movement within the Ni layer during deformation, thereby enhancing strength, but also suppresses the initiation and propagation of microcracks within the TiB_2_ layer.

### 4.2. Analysis of Stress Conditions

When subjected to indentation, the stress state of TiB_2_-Ni multilayer films can be described as follows: Due to its lower yield strength and higher dislocation mobility, the Ni layer undergoes plastic deformation first. The resulting dislocation slip is impeded by the TiB_2_ layer and accumulates at the interlayer interface. The stress concentration caused by dislocation pile-up generates tensile stress *τ_DPU_* in the TiB_2_ layer. This stress induces a tendency for Type I cracking in the TiB_2_ layer, the magnitude of which can be expressed by Equation (2) [23]:

(2) $$\tau_{DPU}=\frac{\pi \left(1-v_{Ni}\right)l{\left(\tau -\tau_{0}\right)}^{2}}{G_{Ni}b}$$

Among these, *G_Ni_* denotes the shear modulus of Ni, *ν_Ni_* represents the Poisson’s ratio of Ni, *l* is the thickness of the Ni layer, and *τ* and *τ*_0_ correspond to the applied shear stress and lattice friction force, respectively.

Additionally, during the indenter’s downward movement, the maximum applied stress *τ_APPL_* perpendicular to the interlayer interface will also act on the TiB_2_ layer. According to the Hall-Petch relationship, the magnitude of *τ_APPL_* will gradually increase as the layer thickness decreases. Furthermore, as the Ni layer undergoes plastic deformation, the resulting work hardening effect will further enhance the magnitude of *τ_APPL_* [24].

### 4.3. Deformation and Fracture Mechanisms

**Figure 13:**
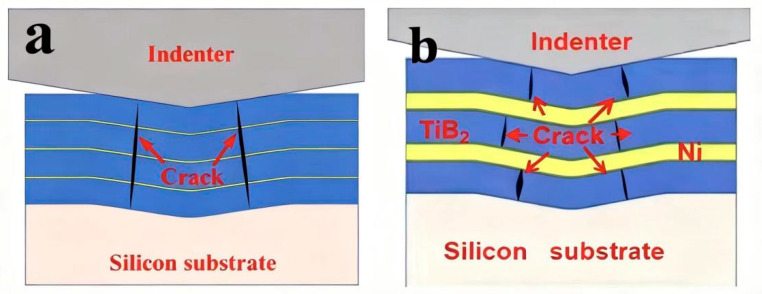
The schematic diagram of deformation of TiB_2_-Ni multilayer films with different Ni layer thicknesses; (**a**) thin nickel films; (**b**) thick nickel fil.

Based on the above analysis, the deformation-fracture mechanism of TiB_2_-Ni multilayer films changes with increasing Ni layer thickness. When the Ni layer is thin, the interfacial stress zone can cover the entire Ni layer, increasing both the activation and slip resistance of dislocations within the Ni layer, thereby elevating the maximum applied stress (*τ_APPL_*). Additionally, according to the Hall-Petch relationship, thinner Ni layers exhibit higher strength. The combined effect of these two factors necessitates a higher *τ_APPL_* for the multilayer film to undergo plastic deformation. The results for pre-yield elastic strain (*H*/*E*) and plastic deformation resistance (*H*^3^/*E*^2^) in Figure 7 further support this conclusion. Thinner Ni layers yield higher *H*/*E* and *H*^3^/*E*^2^ values, indicating superior film elasticity. The higher *τ_APPL_* provides greater energy to the TiB_2_ layer within the multilayer film. Once a crack initiates in the TiB_2_ layer, it propagates rapidly. The thinner Ni layer cannot fully dissipate the energy at the crack tip, allowing the crack to propagate through the entire multilayer film, as illustrated in Figure 13a. When the Ni layer is thicker, the stress zone at the interface cannot fully encompass the entire Ni layer. Furthermore, the relatively low strength of the Ni layer permits localized plastic deformation, which reduces the maximum applied stress (*τ_APPL_*). This alleviates stress concentration at the interface and decreases the energy within the TiB_2_ layer. Moreover, when cracks propagate from the TiB_2_ layer to the interface, the thicker Ni layer substantially hinders crack growth, causing cracks to deflect and propagate within the layer, as illustrated in Figure 13b. The deformation mechanism of the multilayer film involves cracking in the TiB_2_ layer and cooperative plastic deformation in the Ni layer. This shift in deformation and fracture mechanisms is the primary factor contributing to the enhanced toughness of the multilayer film. This transition is also observable in the indentation micrographs (Figure 10). At a Ni layer thickness of 4 nm, numerous step-like cracks surrounding the pit walls are evident. This step-like crack pattern results from the through-cracking behavior of the multilayer film. As the Ni layer thickness increases, these step-like cracks progressively diminish. The primary causes of this phenomenon are the cracking of the TiB_2_ layer and the synergistic plastic deformation of the Ni layer.

### 4.4. Apparent Fracture Toughness and Crack Length Considerations

The calculation of fracture toughness based on Palmqvist crack length measurements derived from surface observations assumes a uniform crack profile through the material thickness. However, extensive research has shown that this assumption is frequently invalid in coating and thin film systems [25]. Even in homogeneous bulk materials, the depth of indentation-induced cracks typically differs from their surface-expressed length. Moreover, Palmqvist-type cracks are not isolated features; instead, they are interconnected via the plastic zone beneath the indenter. This subsurface plastic zone acts as an artificial crack-tip plastic zone, effectively bridging the embedded ends of Palmqvist cracks and thereby blunting their tips [26]. The associated plastic deformation at the indentation site dissipates a portion of the applied energy, leading to shorter subsurface crack extensions compared to surface measurements.

In films exhibiting columnar crystalline structures, compositional gradients, and multiple interfacial layers, such as the TiB_2_-Ni multilayer system, crack propagation is strongly influenced by grain boundaries, residual stresses, and the alternating sequence of soft and hard phases. The resulting three-dimensional crack morphology is significantly more complex, with a high likelihood of crack deflection or arrest at internal interfaces. Therefore, fracture toughness values (*K_IC_*) obtained using this method should be regarded as ‘apparent’ rather than absolute, and are most appropriately used for relative comparisons among specimens tested under identical experimental conditions. Nevertheless, the consistent and significant increase in apparent fracture toughness with increasing Ni layer thickness (Table 2) remains a robust and meaningful indicator of the enhanced resistance to crack propagation in the multilayer film, which is attributable to the microstructural and deformation mechanism changes detailed in Section 4.1, Section 4.2 and Section 4.3.

## 5. Conclusions

The prepared TiB_2_-Ni multilayer films exhibit uniform thickness distribution with well-defined, defect-free interfaces. The Ni layers grow in a columnar grain morphology, whereas the TiB_2_ layers form a fine nanocolumnar structure. With increasing Ni layer thickness, a pronounced (111) texture develops in the Ni layers, accompanied by significant grain coarsening.

Both the hardness and elastic modulus of the TiB_2_-Ni multilayer films decreased with increasing Ni layer thickness. Hardness decreased from 27.3 GPa to 19.3 GPa, while elastic modulus decreased from 247.9 GPa to 228.6 GPa. The fracture toughness of the multilayer films gradually increased from 1.54 MPa·m^1/2^ to 2.73 MPa·m^1/2^. Nanoindentation tests further confirmed that increasing Ni layer thickness simultaneously enhances film-substrate adhesion and overall film toughness. This trend is associated with a transition in deformation behavior from predominantly elastic to increasingly plastic-dominated response.

The improvement in fracture toughness is primarily attributed to a fundamental shift in the deformation–fracture mechanism. In films with thin Ni layers, high interface density and constrained plasticity within the layers lead to elevated stress concentrations, promoting through-thickness crack propagation across the entire multilayer architecture. In contrast, in films with thicker Ni layers, the combined effects of a reduced interfacial stress field and the enhanced capacity of the ductile layers for extensive plastic deformation act synergistically. These mechanisms promote crack deflection at interfaces and energy dissipation within the Ni layers, thereby substantially enhancing fracture resistance.

## Figures and Tables

**Table 1:** Structural parameters of the fabricated TiB_2_-Ni multilayer films.

Deposition Time of Ni/min	Deposition Time of TiB_2_/min	Thickness of Ni Layer/nm	Thickness of TiB_2_ Layer/nm	Number of TiB_2_-Ni Layers	Total Thickness/nm
10	10	50	100	3	450
5	10	25	100	4	500
2.5	10	16	100	4	470
1	10	8	100	5	540
0.5	10	4	100	5	520

**Table 2:** The calculated values of fracture toughness with different Ni layer thicknesses.

Ni Layer Thickness (nm)	*E* (GPa)	*H* (GPa)	*P* (mN)	*a* (μm)	*l* (μm)	*c* (μm)	*K_IC_* (MPa·m^1/2^)
4	247.9	27.3	2000	10.85	9.90	20.57	1.54
8	255.2	25.6	2000	11.03	9.59	20.62	1.70
16	242.7	24.4	2000	11.05	10.38	21.43	1.54
25	236.4	21.8	2000	8.64	7.58	16.22	2.56
50	228.6	19.3	2000	9	7.47	16.47	2.73

## Data Availability

The authors confirm that the data supporting the findings of this study are available within the article.
